# Biological Significance of Urolithins, the Gut Microbial Ellagic Acid-Derived Metabolites: The Evidence So Far

**DOI:** 10.1155/2013/270418

**Published:** 2013-05-28

**Authors:** Juan Carlos Espín, Mar Larrosa, María Teresa García-Conesa, Francisco Tomás-Barberán

**Affiliations:** Research Group on Quality, Safety and Bioactivity of Plant Foods, Department of Food Science and Technology, CEBAS-CSIC, Campus de Espinardo, P.O. Box 164, 30100 Murcia, Spain

## Abstract

The health benefits attributed to pomegranate have been associated with its high content in polyphenols, particularly ellagitannins. This is also the case for other ellagitannin-containing fruits and nuts including strawberry, raspberry, blackberry, walnuts, and muscadine grapes. The bioavailability of ellagitannins and ellagic acid is however very low. These molecules suffer extensive metabolism by the gut microbiota to produce urolithins that are much better absorbed. Urolithins circulate in plasma as glucuronide and sulfate conjugates at concentrations in the range of 0.2–20 **μ**M. It is therefore conceivable that the health effects of ellagitannin-containing products can be associated with these gut-produced urolithins, and thus the evaluation of the biological effects of these metabolites is essential. Recent research, mostly based on *in vitro* testing, has shown preliminary evidence of the anti-inflammatory, anticarcinogenic, antiglycative, antioxidant, and antimicrobial effects of urolithins, supporting their potential contribution to the health effects attributed to pomegranate and ellagitannin-rich foods. The number of *in vivo* studies is still limited, but they show preventive effects of urolithins on gut and systemic inflammation that encourage further research. Both *in vivo* and mechanistic studies are necessary to clarify the health effects of these metabolites. Attention should be paid when designing these mechanistic studies in order to use the physiologically relevant metabolites (urolithins in gut models and their conjugated derivatives in systemic models) at concentrations that can be reached *in vivo*.

## 1. Introduction

The health effects of pomegranate and pomegranate juice have been associated withthe high content in antioxidant polyphenols [1] and particularly in ellagitannins (punicalagins). This is also the case for many other ellagitannin-containing fruits and nuts such as strawberry, raspberry, blackberry, walnuts, and muscadine grapes [2, 3].

Both ellagic acid and ellagitannins have shown relevant biological effects in animal models and human studies which suggests potential preventive effects against chronic diseases such as cancer, diabetes, cardiovascular diseases, and neurodegenerative diseases [4]. These effects are associated with a multitarget action that involves antioxidant, anti-inflammatory, and anticarcinogenic effects [2, 5]. It is well established that ellagitannins and ellagic acid absorption is very low and that the unabsorbed compounds are further metabolized to urolithins by the gut microbiota in the colon [6–10]. This poor bioavailability and the extensive gut catabolism suggest that urolithins rather than ellagitannins or ellagic acid may be the actual bioactive molecules [7, 11]. 

**Figure 1:**
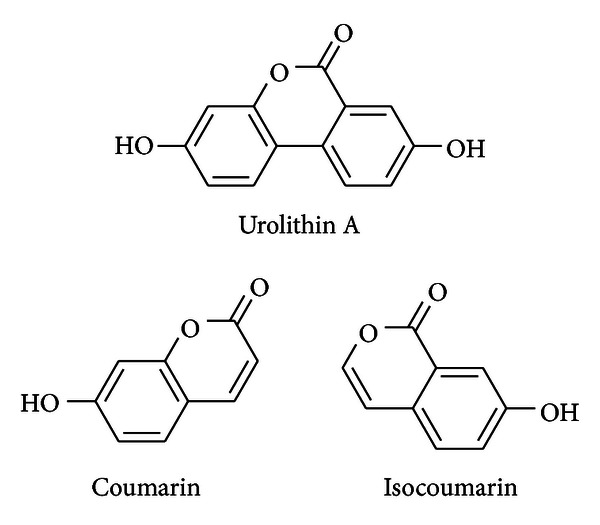
Chemical structure of urolithin A, a benzocoumarin.

**Figure 2:**
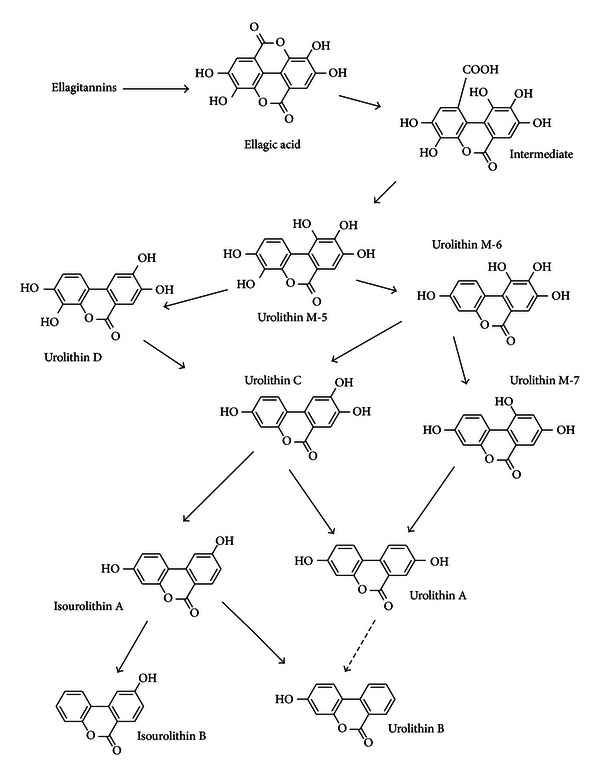
Gut microbiota metabolism of ellagitannins and ellagic acid.

Urolithins are dibenzopyran-6-one derivatives with different hydroxyl substitutions. Chemically they can be considered a combination of coumarin and isocoumarin (benzocoumarins) (Figure 1). They are produced from ellagic acid by the gut microbiota through the loss of one of the two lactones present in ellagic acid (lactonase/decarboxylase activity) and by successive removals of hydroxyls (dehydroxylase activities) [12] (Figure 2). 

Urolithins are not common molecules in nature, but they have been reported in plants rich in ellagitannins, as it is the case of *Tamarix nilotica* flowers [13] and *Punica granatum* leaves that contain urolithin M-5 (3,4,8,9,10-pentahydroxy-dibenzo[*b,d*]pyran-6-one) [14]. They have also been found in some herbivore-derived products, such as the castoreum produced by beavers [15], a product of interest for the perfume industry, and the *Pteropi faeces* (faeces of the squirrel *Trogopterus xanthipes*) that are used in traditional Chinese medicine [16]. Urolithins are also relevant constituents of shilajit, a soil-derived medicinal product used in Ayurvedic medicine [17]. 

The name urolithin was first given to two metabolites isolated from the renal calculus of sheep (*Trifolium subterraneum* has been reported as the cause of clover stone and might be a relevant source of ellagitannins) that were named urolithin A and urolithin B [18]. These two molecules coincided with pigments I and II previously described from castoreum and beaver glands [15]. 

The intake of large amounts of ellagitannins, and particularly of punicalagin, from *Terminalia oblongata* by cattle was associated with hepatotoxicity and nephrotoxicity [19]. However, the subchronic oral administration of large amounts of pomegranate ellagitannins was not toxic to rats where these compounds were found to be extensively metabolized to urolithins [20].

Since urolithins are ellagitannin-derived catabolites that can be absorbed and reach different tissues in the body, they have been suggested as the molecules potentially responsible for the biological effects observed as a consequence of the consumption of pomegranate or other ellagitannin-containing foods [3, 7, 9, 11]. Here we review the state of the art in urolithin metabolites production by gut microbiota, their absorption, tissue distribution and pharmacokinetics, the cell and molecular mechanisms for their biological effects reported so far using different *in vitro* models, and the *in vivo* evidence in animals and humans.

## 2. Urolithins Chemical Nature, Detection, and Identification

Urolithins constitute a whole metabolic family produced by the opening and decarboxylation of one of the lactone rings of ellagic acid and the sequential removal of hydroxyls from different positions (Figure 2). After decarboxylation the first metabolite is urolithin M-5 (pentahydroxy-urolithin), and from this, several tetrahydroxy-urolithin isomers are produced by removal of one hydroxyl group from different positions (urolithin D, urolithin M-6). Trihydroxy-urolithins (urolithin C, urolithin M-7) were then produced after the removal of a second hydroxyl and dihydrox- urolithins (urolithin A and isourolithin A) after the removal of a third one. Monohydroxy-urolithin (urolithin B) was also detected, particularly in those cases in which isourolithin A was produced (Figure 2). Further degradation of urolithins to remove the second lactone ring has not been reported so far, although it should not be discarded. Urolithins show characteristic UV spectra that can be used for the identification of different hydroxyl-substitution patterns on the urolithin nucleus, as well as the further conjugation with methyl, glucuronic acid, or sulfate [21]. The UV spectra of urolithins in methanol exhibit two major absorption bands in the region of 240–400 nm (Figure 4). These are referred to as band I (300–380 nm) and band II (240–280 nm). In many cases, an additional band III (between 280 and 300 nm) is also observed. Unfortunately, bands I and II cannot be associated with a specific ring of the urolithin nucleus [21]. Two groups of urolithin UV spectra were distinguished after a study of the plots of the different spectra: urolithins with a hydroxyl in the 9-position and those without hydroxylation in the 9-position (Figure 4). The occurrence of the hydroxyl at position 9, as is the case of isourolithin A, produces a hypsochromic shift in band I and an increase in the absorption of band II, displaying a maximum around 256 nm, which is the main absorption band of the spectrum of urolithins with a hydroxyl in the 9-position. Conjugation with glucuronic acid or sulfate also has measurable effects on the UV spectrum, and could be used as a diagnostic method for urolithin metabolites identification.

## 3. Bioavailability, Metabolism, and Tissue Distribution of Pomegranate Ellagitannins and Their Metabolites

Due to the potential health effects and the expectation raised by the high antioxidant activity of pomegranate juices that was thought to be due to punicalagins, punicalins and other ellagitannins [1, 22], a sensible step in the research was the determination of the absorption and metabolism of these compounds in animals and humans. The first study testing the bioavailability of pomegranate ellagitannins was carried out on rats, and this showed that after the intake of large amounts of pomegranate husk ellagitannins, the main metabolites detected in plasma and urine were urolithins A, B, and C (Figure 2) and smaller amounts of ellagic acid-dimethyl ether glucuronide [6]. Interestingly, a small amount of punicalagin was also detected in plasma and urine, particularly after a long period of intake, but this has not been confirmed in further studies in humans in which dietary relevant amounts of pomegranate ellagitannins were supplied [7]. 

Urolithin A was produced from ellagic acid, punicalagin, and an ellagitannin-rich walnut extract by fecal microbiota from six volunteers, demonstrating for the first time the production of urolithins by human gut microbiota [12]. In addition, a large interindividual variability was observed in this *ex vivo* experiment [12] in agreement with the results observed *in vivo* [7] suggesting that differences in the microbiota composition affect urolithin production and therefore the potential health effects after consumption of ellagitannin-rich foods [12]. No correlation between urolithin production from ellagitannins and equol production from isoflavones was observed [12], and this indicated that different bacterial strains are involved in the gut metabolism of isoflavones and ellagitannins. 

**Figure 3:**
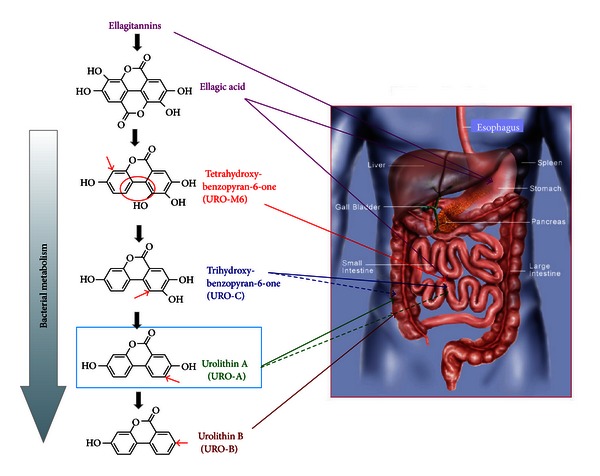
Sequential production of urolithins in the gut.

**Figure 4:**
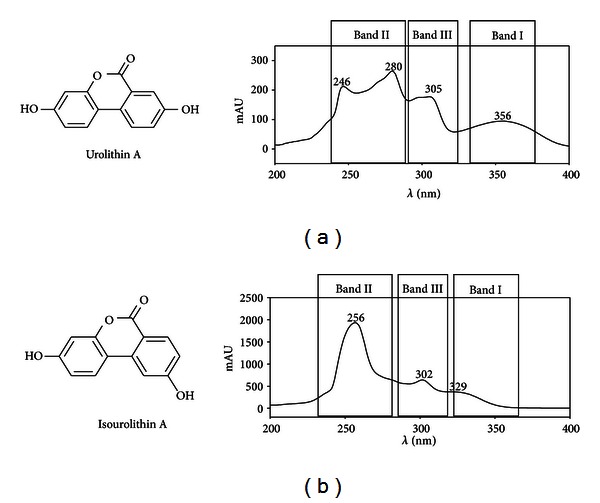
UV characteristic spectra of urolithins.

The Iberian pig was used as a model to study urolithin production from ellagitannins. This particular pig feeds on oak acorns that are very rich in ellagitannins, and this model was used to evaluate ellagitannin metabolism and tissue distribution [23] and to help in understanding the ellagitannin metabolism in humans. This study showed that different urolithins were produced in the gut starting with tetrahydroxy-urolithin through the removal of one of the lactone rings of ellagic acid, and by sequential removal of hydroxyls to end with urolithins A and B (Figure 3). The analysis of plasma and urine samples showed that urolithin A glucuronide and sulfate were the main metabolites, with urolithin C and B glucuronides and sulfates as minor metabolites. Ellagic acid dimethyl ether glucuronide was also a significant metabolite. The analysis of the gall bladder and bile showed that tetrahydroxy-urolithin was absorbed in the first portion of the gut, and was detected in the liver, where it was conjugated and excreted with the bile to the small intestine. An entero-hepatic recirculation was clearly revealed in this animal model study [23]. Regarding the tissue distribution, urolithin metabolites only accumulated in the urine bladder and the gall bladder where they reached high concentrations, but they did not accumulate in any of the analyzed tissues (muscle, adipose tissue, kidney, liver, heart, etc.) [23]. 

The occurrence of low concentrations or urolithin metabolites in mouse prostate gland after pomegranate ellagitannins intake [24] and in human prostate after the intake of pomegranate juice and walnuts [25] has been reported. This last study is the only one available on the evaluation of the occurrence of ellagitannin metabolites in human tissues (prostate biopsies), and the occurrence of urolithin A glucuronide (2 ng/g tissue) and traces of urolithin B glucuronide and ellagic acid dimethyl ether was reported [25].

There are no studies on the pharmacokinetics and tissue distribution of urolithins after their direct intake in humans. There is, however, indirect evidence of both aspects after the intake of ellagitannins or ellagic acid-rich food products. In the case of pomegranates or derived beverages, it is well established that ellagitannins are not absorbed when dietary doses are ingested and that the released ellagic acid is absorbed in the first part of the gastrointestinal tract and is detected as such in plasma with *C*_max⁡⁡_ concentrations around 100 nM with *T*_max⁡⁡_ 1 hour after the intake [10, 26]. Ellagic acid conjugates have also been detected, and these include methyl ether and glucuronyl and sulphate conjugates. The most common metabolite found in urine and plasma is ellagic acid dimethyl ether glucuronide, which involves the methylation by COMT and then the glucuronidation by glucuronyl-transferase [3, 7]. The peak plasma levels of urolithin A were 14–25 *μ*M depending on the volunteers. These metabolites started to appear in plasma 6–8 hours after the intake, which confirms urolithin production in the colon [7], and persisted in urine and plasma 48–72 hours after the ellagitannins intake showing an active enterohepatic recirculation [3, 7, 9, 12].

Seeram et al. [24] described the pharmacokinetics and tissue distribution of urolithins in mice after oral or intra-peritoneal administration of chemically synthesized urolithin A (human equivalent dose, HED ~85 mg for a 70 kg person). Urolithin A peaked in plasma after 2 h and reached the highest concentrations in the prostate followed by the small intestine and colon, with a peak at 4 h [24]. Urolithin A, urolithin A sulfate, and methyl-urolithin A were mainly detected in the prostate gland whereas urolithin A glucuronide was primarily detected in liver and kidney tissues. Other studies with animal models, including rats and pigs, confirmed the urolithin production in the colon, and the sequential loss of hydroxyls when the metabolites advance in the intestinal tract [3, 12]. 

González-Sarrías et al. [25] reported the occurrence of urolithin metabolites and ellagic acid dimethyl ether glucuronide in prostate biopsies after the intake of pomegranate juice. This is the only study carried out so far to evaluate the occurrence of urolithin metabolites in human tissues. The concentration detected in the prostate gland was however low, although higher concentrations should not be discarded, as suggested by a parallel study carried out in rats [25] and also in agreement with the results obtained by Seeram et al. [24].

## 4. Production of Urolithins by Different Animals

The production of urolithins from ellagitannins has been reported in different animals (Table 1). Those animals that feed on bark and wood, as is the case of beavers and squirrels, produce urolithins in their gut, and they are present in their feces and in excretions/secretions as is the case of castoreum.

In addition, ruminants that feed on ellagitannin-rich fodders (oak leaves, *Trifolium subterraneum*, etc.) also produce urolithins that circulate in plasma as conjugated derivatives and are excreted in urine and feces. The production of urolithins in the rumen of cattle has been demonstrated, and isourolithin A and urolithin B were the main metabolites observed, while urolithin A seems to be mainly produced in the intestine [31]. The kinetics of the production of the different metabolites suggests that urolithin B production from isourolithin A is more favored than its production from urolithin A that seems to be an end product. The occurrence of urolithins in milk seems likely, although it has not been demonstrated so far. 

In monogastric animals, urolithins are produced in rat, mouse, and pig and in all these cases the main metabolite present in feces, urine, and plasma is urolithin A and its glucuronide and sulfate conjugates. Urolithins C and B are less frequent, but they have also been detected together with small amounts of isourolithin A (Table 1). The same behavior is observed in humans after the intake of pomegranates, walnuts, tea, muscadine grapes, strawberries, raspberries, blackberries, cloudberries, oak acorns, and oak-aged red wine. Urolithins will also be eventually produced after the intake of all ellagitannin-containing foods and medicinal plants as is the case of camu camu (*Myrciaria dubia*), arctic bramble, rose hip, sea buckthorn, cranberry, *Geranium*, and oak-aged spirits (whisky, etc.).

The studies to evaluate urolithin production in other animals, including birds and insects, are very limited [21], but the evidence so far indicates that they do not produce urolithins from ellagitannins. In insects, ellagitannins are hydrolyzed to release ellagic acid, which is detected in feces. This was shown in the acorn beetle (*Thorectes lusitanicus*) [21]. Indirect evidence shows that honeybees harvesting nectar containing ellagitannins hydrolyze them to release ellagic acid during honey maturation [37], but no urolithins are detected suggesting that honeybee microbiota is not able to metabolize ellagic acid to produce urolithins. In termites feeding on wood, ellagitannins are hydrolyzed to ellagic acid, and then hydroxyls are removed to produce nasutins, and it seems that termite microbiota does not have the ability of removing the lactone ring of ellagic acid to produce urolithins [21]. It has been reported that the hemolymph of some Australian termites of the genus *Nasutitermes* contains nasutin A that is also present in feces, but no urolithin was detected [38]. 

In birds the only study available was done with greenfinches (*Carduelis chloris*) that were fed for 2 weeks with blackberries. Feces were collected and analyzed, and only ellagic acid was detected [21].

These results show that urolithins are generally produced by mammals after the intake of ellagitannins. There is, however, interindividual variability that has been suggested to be associated with different gut microbiota composition, and this means that the health effects observed after the intake of pomegranates and other ellagitannin-containing food can be modulated by the occurrence of specific microbiota to produce urolithins [50, 51]. 

## 5. Biological Activity of Urolithins

The bioavailability and metabolism studies clearly indicate which metabolites should be tested and at what concentrations in the mechanistic studies to understand the biological activity of pomegranate ellagitannins. A combination of punicalagin, ellagic acid and urolithins should be tested in those studies using models of gastrointestinal tract cells where they can reach concentrations around several hundred *μ*M (for punicalagin and ellagic acid) and tens *μ*M for urolithins. 

The health effects attributed to urolithins based on studies carried out *in vitro* are numerous and diverse, from antimalarial properties or topoisomerase inhibitors to quenchers of bacterial quorum sensing. In general, the number of publications regarding each biological activity is still very limited, although these studies are generally carried out using a suitable physiological concentration range, in the order of submicromolar-low micromolar concentrations, similar to those that urolithins can reach in the gut and plasma after the ingestion of ellagitannin-rich foods (Table 2). From all the studies carried out *in vitro*, it is important to highlight a recent study in which the biological effects of urolithin glucuronides, the major circulating metabolites in plasma after ingestion of ellagitannin-rich foods, were explored in human aortic endothelial cells [45]. This type of study is still very scarce, although highly desirable, when studying the mechanism of action of polyphenols or their metabolites outside the gastrointestinal tract, as polyphenols and their gut microbiota metabolites are glucuronidated by Phase II enzymes after absorption, and they are found in this conjugated form in the circulatory system and peripheral tissues.

### 5.1. Antioxidant Activity

Given the high antioxidant properties of ellagitannin-rich foods, as is the case of pomegranate [1], one of the first activities that had been explored for urolithins was their antioxidant capacity. The first available study reported that urolithin A had an antioxidant capacity 42-times lower than that of its precursor punicalagin when it was tested in the DPPH assay and 3,500 times lower when it was tested in the ABTS assay [7]. These results were consistent with the concentrations of urolithins necessary to reach an IC_50_ in several antioxidant assays (DPPH, xanthine-XOD, and PMS-NADH) that were above 100 *μ*M [29]. However, using the ORAC assay, all urolithins tested exhibited potent antioxidant properties compared with those of ascorbic acid, urolithin A being the most potent among them [29]. In a similar study, in which the antioxidant activity of a large number of polyphenols and their metabolites produced *in vivo* was compared by the ORAC method, the antioxidant activity of urolithin A was one of the most powerful, only beaten by some proanthocyanidin oligomers, catechin, epicatechin and 3,4-dihydroxyphenyl acetic acid [52]. Using a cell-based method, in which the transport through the cell membrane was considered, Bialonska et al. [39] determined that urolithin C showed the highest antioxidant power (IC_50_ = 0.16 *μ*M) whereas urolithin A showed an IC_50_ of 13.6 *μ*M, still in the range of the plasma concentrations achievable *in vivo* but in a lesser degree than its precursor ellagic acid (1.1 *μ*M) or vitamin C (1.9 *μ*M) [39]. In neuronal cells in which an oxidative stress was induced, urolithin B (0.5–20 *μ*M) and urolithin A (10 *μ*M) exhibited a protective effect increasing cell survival [40]. In general, it is assumable that urolithins have higher antioxidant capacity than was originally thought but the antioxidant capacity depends on the measurement method and needs to be studied in more detail and with other *in vivo* methods given the importance of free radicals in many diseases.

### 5.2. Estrogenic Modulators

In the last few years, there has been an increasing interest in the study of the estrogenic/antiestrogenic activity of plant-derived compounds (phytoestrogens) due to their potential benefits as part of the diet, that is, regulation of cholesterol levels or maintenance of the bone density after menopause. Many polyphenols (isoflavones, flavanones, estilbenes, etc.) show phytoestrogenic effects. In some cases, dietary polyphenols may be the precursors of the so-called enterophytoestrogens that are produced by the colon microbiota by catabolism of the original phenolics. The potential activity of urolithins as enterophytoestrogens has recently been investigated [41]. In this work, structure-activity studies revealed that the urolithins A and B had specific molecular characteristics that made these molecules potentially able to bind with the *α*- and *β*-estrogenic receptors (ER) [41]. The ER Competitive Binding Assays showed that both urolithins had an affinity for the ER*α* and ER*β* receptors, that urolithin A bound more effectively than urolithin B, and that the affinity was higher for the ER*α* than for the ER*β* receptor. Using a proliferation assay with cells sensitive to estrogens (E-screen) urolithins A and B exhibited estrogenic activity (in the absence of estradiol) and antiestrogenic activity (in the presence of estradiol) in a similar fashion to other known phytoestrogens [41].

### 5.3. Anti-Inflammatory-Related Activities

#### 5.3.1. Antimalarial Activity

In an attempt to clarify whether the properties of the sun-dried rind of immature *Punica granatum*, that is, used as an anti-malarial herbal remedy, are at least in part due to urolithins, their activity inhibiting the MMP-9 enzyme (directly involved in the pathogenesis of malaria) was tested. Urolithins A and B at concentrations of 25 *μ*M inhibited the release of MMP-9 and its mRNA expression, in hemozoin and TNF-*α* stimulated monocytic cells [42]. These results indicated that the antimalarial properties of pomegranate rind may partly be due to urolithins.

#### 5.3.2. Histone Acetylation Status

Histone acetylation/deacetylation status plays an important role in inflammation since it is associated with the activation/deactivation of transcription factors as NF-*κ*B and AP-1, directly implicated in inflammation. One of the mechanisms by which urolithins exert their anti-inflammatory activity could be the inhibition of histone acetyltransferases (HAT) as in fact has been demonstrated for 5 *μ*M urolithin A and B that were able of inhibiting HAT activity [53].

#### 5.3.3. Anti-Inflammatory Effects on Human Colon Fibroblasts

Colon fibroblasts have an important role in the gut immune response and can be exposed to significant quantities of colon dietary metabolites. Following ellagitannins intake, urolithin A and urolithin B as well as traces of ellagic acid can be found in the intestine. Thus, cultured human colon fibroblasts stimulated with proinflammatory cytokines were exposed to a mixture of urolithins A and B and ellagic acid at concentrations representative of those that may be found *in vivo* (5–40 *μ*M) [43]. Urolithin A and, most significantly, the mixture of metabolites were able to inhibit the migration capacity of the fibroblasts and the adhesion of monocytes to the fibroblasts giving evidence of a potential amelioration of inflammation in the colon cells [43]. Further molecular insights into these responses revealed that these effects were concomitant with a significant downregulation of the levels of prostaglandin-E2 (PGE2), PAI-1, and interleukin 8 (IL-8) as well as of other key regulators of cell migration and adhesion (Table 2). The study of the individual metabolites indicated that urolithin A was the most active compound. In addition to the inhibition of PGE2, urolithin A and also urolithin B were able to inhibit the expression of the two major enzymes responsible for the synthesis of prostaglandins under inflammatory conditions (mPGES-1 and COX-2) whereas ellagic acid did not show any effect [44]. The anti-inflammatory effects of urolithins may be mediated through regulation of the transcription factor NF-*κ*B since both urolithins are able to inhibit the activation of this factor and also exhibit an inhibitory effect on the mitogen-activated protein kinase pathways (MAPK pathways), c-Jun (urolithin A), and p38 (urolithins A and B) [44].

#### 5.3.4. Anti-Inflammatory Effects on Human Endothelial Cells

The anti-inflammatory effects of urolithin glucuronides have recently been investigated by Giménez-Bastida et al. [45]. Using cytokine-induced human aortic endothelial cells, this study explored the effects of physiologically relevant concentrations (low *μ*M range, 1–20 *μ*M) of urolithin A and B glucuronides (the main metabolites detected in human plasma after the oral intake of ellagitannin-containing foods [7]) on two early atherosclerotic events: monocyte adhesion to endothelial cells and endothelial cell migration. The main outcome of this research was that urolithin A glucuronide, the most abundant circulating conjugate after the intake of ellagitannins, exhibited the highest anti-inflammatory activity. Urolithin A glucuronide moderately but significantly inhibited monocyte adhesion to the endothelial cells as well as the migration capacity of these endothelial aortic cells [45]. These effects were associated with a moderate but significant regulation of the levels of several key molecular markers associated with the atherosclerotic process: (i) downregulation of chemokine (C-C motif) ligand 2 (CCL2), (ii) downregulation of plasminogen activator inhibitor-1 (PAI-1), and (iii) regulation of several growth factors. These results were, in some cases, comparable to those observed for the corresponding aglycone urolithin A and suggested that the glucuronidation of urolithin A does not entirely eliminate the activity of the aglycone and that the glucuronide may contribute to the beneficial effects against cardiovascular diseases attributed to the consumption of pomegranate (or pomegranate juice) [22] and other ellagitannin-containing foods [2].

### 5.4. Anticarcinogenic-Related Activities

#### 5.4.1. Topoisomerase II and CK2 Inhibitors

The anticarcinogenic activity of urolithins is one of the most explored so far. Urolithins seem to exert anticarcinogenic activity by affecting numerous molecular pathways [5]. Urolithins are inhibitors of the CK2 enzyme, a ubiquitous protein kinase implicated in a wide variety of cell functions that when altered lead to processes like inflammation and cancer. Using submicromolar concentrations in *in silico* screening, urolithin A was discovered to be a potent and selective CK2 inhibitor (IC_50_ = 0.39 *μ*M) [54]. Urolithin M5 and another synthetic urolithin (never detected as an ellagic acid or ellagitannin metabolite in animals) showed topoisomerase inhibitory activity at concentrations below 1 *μ*M, showing an even more potent activity than that of the chemotherapeutic drug doxorubicin. The molecular mechanism of action toward human topoisomerase II seems to be the competition with ATP for the ATP binding pocket of the human enzyme [55].

#### 5.4.2. Human Prostate Cells

The human prostate gland is one of the organs where urolithins can be detected after the consumption of pomegranate juice and walnuts [25]. In prostate cancer cells, urolithins (A, B, C, and D) inhibited CYP1B1 activity (a target in prostate cancer chemoprevention) in a dose ranging from 1.15 *μ*M (urolithin A) to 137 *μ*M (urolithin D) whereas higher concentrations were needed for CYP1A1 activity inhibition (12.4–2,907 *μ*M). The changes in *V*_max⁡⁡_ and *K*_*m*⁡_ parameters with increasing concentrations of CYP1B1 inhibitor suggested an uncompetitive inhibition for urolithin A. The lack of changes for these parameters, however, suggested a noncompetitive inhibition for urolithin B. Furthermore, the decrease in CYP1B1 activity exerted by urolithins was accompanied by a decrease in CYP1B1 expression [46].

#### 5.4.3. Anticancer Effects against Human Colon Cancer Cells

The *in vitro* studies conducted in colon cancer cells are of great relevance since it is in this portion of the GI tract where urolithins are produced and can reach bioactive concentrations. A mixture of urolithin A, urolithin B, and ellagic acid, at concentrations representative of those attainable in the intestine through the diet, inhibited the proliferation of the human colon cancer Caco-2 cells [47]. This inhibition was mostly mediated through an S and G_2_/M cell cycle arrest in association with the modulation of the expression of genes involved in cell cycle regulation (*CCNB1* and *CCNB1IP1*) and in cancer development, such as the oncogenes *K-Ras* and *c-Myc*, the tumor suppressors *DUSP6* and *Fos,* and the growth factors receptors *FGFR2* and *EGFR* [47]. These results are concomitant with a general regulation of the ERK1/2 signaling pathway. Another pathway that may be a target for urolithins is the Wnt pathway. Using a luciferase reporter of the canonical Wnt pathway in HEK T293 colon cells, Sharma et al. [48] showed that urolithin A was able to inhibit Wnt signaling with an IC_50_ of 39 *μ*M whereas ellagic acid showed an IC_50_ of 63 *μ*M, concentrations that can be achieved in the colon after pomegranate or ellagitannin-rich foods consumption [48]. Kasimsetty et al. [49] also tested and compared the effects of urolithins on human colon cancer HT-29 cells, and found that urolithins A, B, C, and D were able to induce apoptosis in a concentration range between 25 and 50 *μ*M, whereas much higher concentrations (~500 *μ*M) were necessary to promote cell cycle arrest. Urolithins A, B, C, and D also inhibited CYP1 activity (by approximately 50%) using concentrations in the range of 50 to 75 *μ*M. All these results suggest that regular dietary consumption of ellagitannin-containing foods, which can yield and maintain *μ*M concentrations of urolithins in the colon, may have a protective effect against colon cancer development. 

### 5.5. Protein Glycation Inhibitors

The production of advanced glycation end products is a secondary effect of hyperglycemia that has a significant role in the cardiovascular complications associated with diabetes and also with Alzheimer's disease. Urolithins A and B (1 *μ*M) showed significant antiglycative activity that increased when increasing the concentrations up to 10 *μ*M in the case of urolithin A. The same increase was not observed for urolithin B [40]. This activity, however, was not related to their antioxidant activity (measured as ABTS) or to their glyoxal-binding capacity.

### 5.6. Antimicrobial Activity through the Inhibition of Quorum Sensing

Quorum Sensing (QS) is a bacterial communication mechanism that responds to small molecules or auto-inducers and by which bacteria are able to detect population density, regulate gene expression, and control several key processes related to the infection progression such as virulence, biofilm formation, and motility. An important area of recent research focuses on finding natural compounds able to inhibit QS that may pose an efficient alternative to the use of antibiotics against pathogen infections. Dietary-derived polyphenol metabolites present in the intestine may contribute to reduce the ability of pathogens to invade the intestine through inhibition of their QS capacity. Urolithins A and B have been shown to reduce biofilm biomass and swimming motility of the enteropathogen *Yersinia enterocolitica *at concentrations as low as 4 *μ*M [56]. These effects were associated with a significant reduction of the levels of the bacteria autoinducers acylhomoserine lactones (AHLs) released by the bacteria to the culture media and were accompanied by the alteration of the expression levels of genes critically involved in the synthesis of lactones (*yenI* and *yenR*) and the synthesis of the flagella (*flhDC*, *fliA,* and *fleB*) [56]. These results suggest that the microbiota-derived metabolites urolithin A and urolithin B may exert antipathogenic effects in the colon against *Y. enterocolitica* and may contribute to maintain the microbial equilibrium in the gut.

## 6. The *In Vivo* Evidence

The poor bioavailability of ellagitannins, and ellagic acid derivatives as well as their extensive metabolism in the gastrointestinal tract has raised the question whether these parent molecules, found as such in the food, were the real active compounds systemically that could be related to the health benefits exerted by pomegranate juice and other ellagitannin-containing foodstuffs. Our group reported for the first time the occurrence of urolithins in humans after consuming pomegranate juice [7]. These metabolites reached micromolar concentrations in the bloodstream, and we launched the hypothesis that linked the potential systemic biological effects of pomegranate juice ingestion with urolithins rather than with the polyphenols present in pomegranate juice [7] or other ellagitannin-containing foodstuffs such as walnuts, strawberries, and raspberries [8]. 

The first direct *in vivo* evidence regarding the biological activity of urolithins was also reported by our group five years later after launching the above hypothesis. Larrosa et al. [29] reported the anti-inflammatory and prebiotic effects of the most significant urolithin metabolite (urolithin A) in a rat model of ulcerative colitis. The rats received a standard chow supplemented either with 250 mg/kg/day of an ellagitannin-rich pomegranate extract (PE) (HED ~2.5 g in a 70 kg person) or with 15 mg/kg/day of synthetic urolithin A (UroA) (HED ~154 mg in a 70 kg person) for 25 days before inducing colon inflammation with dextran sodium sulfate (DSS). The pomegranate extract contained 35% punicalagins, 13% punicalin, 4.5% ellagic acid glycosides, and 8.9% free ellagic acid. Taking into account that urolithin A is the main metabolite produced after pomegranate intake, the objective of that study was to evaluate a possible direct effect of urolithin A ingested as synthetic compound and to compare this effect with that exerted by the *in vivo* generated urolithin A after pomegranate extract consumption. A number of measurements were carried out, including the evaluation of colon tissue damage, microbiota changes, antioxidant status, prostaglandin E2 (PGE2), nitric oxide production, cyclooxygenase-2 (COX-2), inducible nitric oxide synthase (iNOS), prostaglandin E synthase (PTGES), gene expression in colon mucosa (microarrays and RT-PCR) and polyphenol metabolism (LC-MS-MS). The administration of both pomegranate extract and urolithin A for 25 days before the induction of inflammation was reported to be safe according to serobiochemical analysis and evaluation of the animals. Both pomegranate extract and urolithin-A decreased the anti-inflammatory markers iNOS, COX-2, PTGES and PGE2 in colonic mucosa. However, the anti-inflammatory activity exerted by 15 mg/kg urolithin A at both colonic and systemic levels was relatively stronger than that produced by 250 mg/kg pomegranate extract. Only urolithin A was able to preserve the colonic architecture. However, pomegranate extract but not urolithin A decreased oxidative stress in plasma and colon mucosa (TBARs and FRAP methods). 

**Figure 5:**
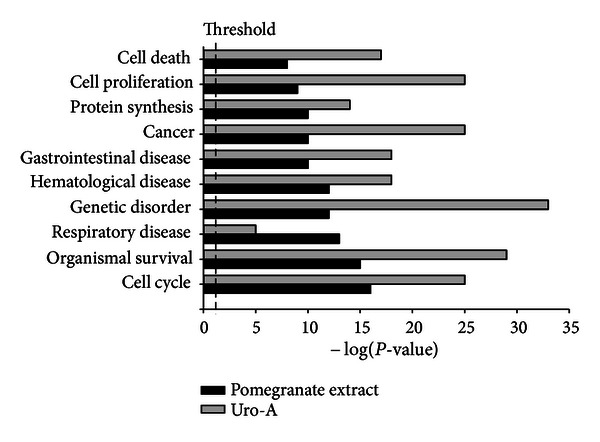
Common top ten functions and diseases identified by functional analysis (IPA, Ingenuity Pathways Analysis; Ingenuity Systems, Redwood City, CA, USA) and that were most significant to gene expression altered by the intake of pomegranate extract or Urolithin A in the colon mucosa of a rat model of ulcerative colitis (gene expression data from Larrosa et al. [29]). Black bars: pomegranate extract. Grey bars: urolithin A. A Fischer's exact test was used to calculate a *P*-value determining the probability that each function or disease assigned to the data was due to chance. We set a threshold at *P* value ≤ 0.05 (dashed line) which corresponds to a False Discovery Rate (FDR) ≤ 5% of false positives. Note: On the *y*-axis, the significance is expressed as the minus log (10-based) of the *P* values. The higher the bar, the lower the *P* value and hence the more significant for the enrichment of the function/disease.

Regarding gene expression analyses in the colon mucosa of a rat model of ulcerative colitis, the expression of 2,058 and 6,996 genes was found to be significantly modified by the consumption of the pomegranate extract and urolithin A, respectively. From those, a total of 667 genes were commonly regulated both by the pomegranate extract and urolithin A. These genes were used for Functional Analyses using the Ingenuity Pathways Analysis (IPA, Ingenuity Systems, Redwood City, CA, USA).  Figure 5 shows the main top functions and diseases that were most significant to the gene expression altered by the intake of the pomegranate extract or urolithin A. Of note, some of the main functions and disorders detected were associated to colon cancer development, for example, “cell death,” “cell proliferation,” “cancer,” “gastrointestinal disease,” “organismal survival,” and “cell cycle.” 

**Figure 6:**
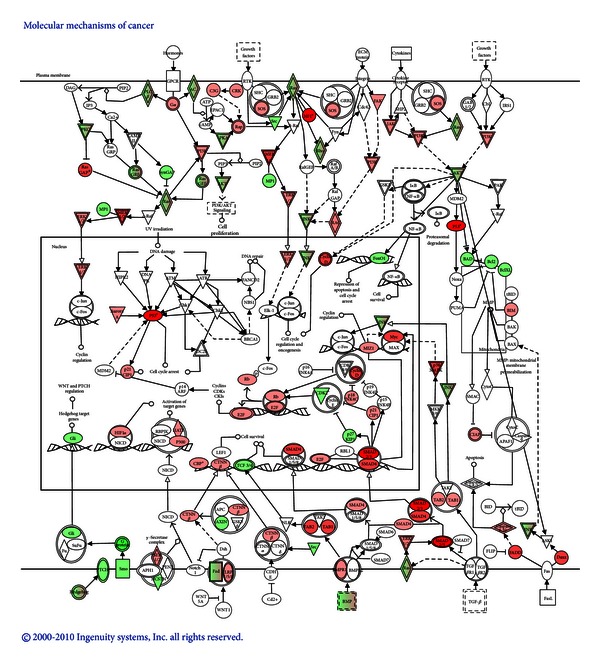
Graphical representation of main genes involved in signaling pathways, transcription regulation, cell growth, cell cycle, and apoptosis for which deregulation has been implicated in cancer development. Genes depicted in red color were upregulated and genes represented in green color were downregulated in the colon mucosa of rats following the consumption of urolithin A, (Larrosa et al. [29]). Analyses were carried out using the Canonical Pathways Analysis tool of Ingenuity Pathways Analysis (Ingenuity Systems, Redwood City, CA, USA).

Using the Canonical Pathways Analysis tool of IPA, it was also possible to identify the main pathways from the IPA library that were most significant to the gene expression data analyzed.  Figure 6 shows the main molecules specifically involved in signaling pathways, transcription regulation, cell growth, cell cycle, and apoptosis for which deregulation has been implicated in cancer development and whose transcript levels were significantlymodulated by urolithin A (upregulated in red, downregulated in green). The different molecules are depicted in their corresponding cellular compartment, and reported interactions between them are indicated by arrows. This figure illustrates the complexity of the molecular mechanisms that may be triggered in a cell as a response to the exposure to a potential bioactive compound, for example, urolithin A. It should be noted that among the changes detected, some important tumor suppressors such as p53 and Rb1 were upregulated whereas the antiapoptotic genes Bcl_XL_ and Akt were downregulated by urolithin A. These changes were also observed after treatment with the pomegranate extract. These results are indicative of *in vivo* molecular modulation in the colon mucosa cells in response to urolithin A or pomegranate extract that may be associated with the prevention or reduction of malignant changes and cancer development. In support of these results, *in vitro* studies using human colon cancer cells Caco-2 also reported the inhibition of these cells proliferation and a cell cycle block by urolithin A (Table 2). In addition, many of those genes involved in these functions were found to be modulated by urolithin A in the human cells [47]. 

Our group also reported for the first time the *in vivo* prebiotic effect of pomegranate extract and urolithin A [29]. Both pomegranate extract and urolithin A modulated favorably the gut microbiota in healthy rats (before inflammation) which could contribute to the protective effects of pomegranate juice and urolithin A against the further induced colon inflammation. In the case of urolithin A, the effect was unequivocally due to synthetic metabolite orally administered and could be associated to the inhibition of some specific gut microbiota species to favor the growing of some (beneficial) microbial groups such as lactobacilli and bifidobacteria. In the case of pomegranate extract, the same explanation could be also given, but a prebiotic effect exerted by other compounds present in the extract (fibers, sugars, etc.) could not be ruled out [29]. 

The second study dealing with the *in vivo* evidence of urolithins was carried out by Ishimoto et al. [57]. These authors reported the acute (24 h) anti-inflammatory effect of urolithin A on carrageenan-induced paw edema in mice. In this case, urolithin A (300 mg/kg; HED ~1.5 g in a 70 kg person) was orally administered to mice at 1 or 6 h before the injection of carrageenan. The inflammatory effect was evaluated for 24 h after carrageenan injection by measuring the hind paw volume. Unfortunately no inflammatory markers were measured in this study. The authors claimed a rapid absorption of urolithin A which peaked at 1 h in mice [57]. However, this was not in agreement with a previous study dealing with the pharmacokinetic and tissue distribution of urolithins where urolithin A peaked in plasma after 2 h, being almost undetectable after 6 h [24]. Ishimoto et al. [57] described a high antioxidant activity of urolithin A in mouse plasma using the ORAC method and suggested that this activity could be correlated with the anti-inflammatory effects. These results were not in agreement with those of Larrosa et al. [29] who suggested that anti-inflammatory effects of urolithin A were not mediated by its antioxidant activity since no significant antioxidant effects in plasma and colon of rats were found (TBARs and FRAP methods). In addition, Cerdá et al. [7] did not observe any increase in the antioxidant status of human plasma after the intake of 1 L of pomegranate juice for 5 days (ABTS and DPPH methods). Perhaps, the high dose of urolithin A assayed by Ishimoto et al. [57] as well as the specific use of the ORAC method to measure the antioxidant activity could be behind this apparent controversy. Overall, the human extrapolation of the results obtained by Ishimoto et al. [57] could be doubtful due to their assay conditions. These authors assayed a single dose of 1.5 g HED of urolithin A which raises safety concerns in contrast to the assay conditions approached by Larrosa et al. [29].

## 7. Outlook and Further Research

Urolithins can reach significant concentrations in the human body after the intake of ellagitannin-containing products; however, the direct biological activity of urolithins has been scarcely studied so far. In this regard, these metabolites could be the missing link to explain the health benefits associated to the consumption of ellagitannin-containing products such as pomegranate. There are a number of urolithins produced by the human gut microbiota. Some studies reveal the relevant turnover of these metabolites in the human body, with a high urine excretion, significant concentration in the bloodstream, and also disposition in the human prostate gland. To date, only two studies (animal models) have described the *in vivo* anti-inflammatory [29, 57] and possible cancer chemopreventive activities [29] of the most abundant urolithin produced in humans, urolithin A. In addition to this limited evidence, the direct *in vivo* activity of the rest of urolithins is not yet known, and thus more animal studies are needed. *In vitro* studies with specific cell models and specific metabolites (type of urolithin and conjugates such as glucuronides or sulfates) are useful to unravel mechanisms. In this context, appropriate concentrations and type of metabolites should be carefully chosen to draw relevant conclusions from these *in vitro* models. 

The evaluation of urolithins in humans could be more difficult. Although these metabolites are produced in the human gut, the administration of synthetic urolithins to humans can raise safety concerns since these metabolites are not found as such (at least in relevant concentrations) in foodstuffs, and therefore they cannot be considered as dietary compounds. In this regard, the toxicological evaluation of urolithins in animal models is also missing.

## Figures and Tables

**Table 1:** Production of urolithins as ellagitannin metabolites in different animals and humans.

Animal production	Ellagitannin source	Metabolites	References
Rat (*Rattus norvegicus*)	Pomegranate husk	Uro A, Uro B, Uro C	[6, 20]
Rat (*Rattus norvegicus*)	Ellagic acid	Uro A	[27]
Rat (*Rattus norvegicus*)	Oak-flavored milk	Uro A, Uro B, Uro C	[28]
Rat (*Rattus norvegicus*)	Pomegranate extract	Uro A	[29]
Rat (*Rattus norvegicus*)	Geraniin (from *Geranium thunbergii*)	Uro A, Uro M-6, Uro M-7, Uro M5	[30]
Mouse (*Mus musculus*)	Pomegranate extract	Uro A	[24]
Mouse (*Mus musculus*)	Pomegranate husk	Uro A	[21]
Beaver (*Castor canadensis*)	Wood	Uro A; Uro B	[15] [21]
Complex-toothed squirrel (*Trogopterus xanthipes*)	Unknown	Uro A	[16, 21]
Sheep (*Ovis aries*)	*Trifolium subterraneum *	Uro A, Uro B	[18]
Sheep (*Ovis aries*)	Quebracho	Uro A	*
Cattle (*Bos primigenius*)	Young oak leaves	Uro A, IsoUro A, Uro B	[31]
Pig (*Sus scrofa domesticus*)	Acorns	Uro A, Uro C, Uro D, Uro B	[23]
Humans (*Homo sapiens*)	Pomegranate juice	Uro A, Uro C, Isouro A, Uro B	[7, 9, 25, 32]
Humans (*Homo sapiens*)	Pomegranate extract	Uro A, UroB, Uro C	[10]
Humans (*Homo sapiens*)	Walnuts	Uro A, Uro B, Uro C	[8] [25]
Humans (*Homo sapiens*)	Strawberry	Uro A, Isouro A, Uro B, Uro C	[8, 33]
Humans (*Homo sapiens*)	Raspberry	Uro A, IsoUro A, Uro B	[8, 34]
Humans (*Homo sapiens*)	Blackberry	Uro A, Uro C	*
Humans (*Homo sapiens*)	Cloudberry	Uro A	*
Humans (*Homo sapiens*)	Oak-aged red wine	Uro A	[8]
Humans (*Homo sapiens*)	Tea	Uro A	[35]
Humans (*Homo sapiens*)	Nuts	Uro A, Isouro A, Uro B	[36]

Uro A (urolithin A); Uro B (urolithin B); Uro C (urolithin C); Uro D (urolithin D); Isouro A (isourolithin A); Uro M-5 (urolithin M-5); Uro M-6 (urolithin M-6); and Uro M-7 (urolithin M-7). *Tomás-Barberán et al., unpublished results.

**Table 2:** Biological effects of urolithins assayed on human cell lines.

Test compound	Activity	Test model	Dose/duration	Effect	Ref.
UA, UB, UC, UD	Antioxidant	PMA DCFH-DA HL-60 cells	0.04–137 µM, 0.5 h	Inhibit cellular injury caused by ROS	[39]
UA, UB	Antioxidant	DMNQ SK-N-MC cells	0.1–20 *μ*M, 0.5 h	Increase cell survival	[40]
UA, UB	Estrogenic Anti-estrogenic	MCF-7 cells	0.1–40 *μ*M, 7 d	Induction of cell proliferation (estrogenic)/inhibition of cell proliferation in the presence of estradiol (anti-estrogenic)	[41]
UA, UB	Antimalarial	THP-1 stimulated with haemozoin or TNF-*α*	25 *μ*M, 48 h	Inhibit the release and expression of MMP-9	[42]
UA, UB	Anti-inflammatory	CCD18-Co/THP-1 stimulated with TNF-*α*	5–40 *μ*M, 48 h	Decrease fibroblasts migration and monocyte adhesion to fibroblasts	[43]
UA, UB	Anti-inflammatory	CCD18-Co stimulated with IL-1*β*	0.1–40 μM, 18 h	Inhibit PGE2 production and mPGES-1 and COX-2 expression	[44]
UA, UB, UAG, UBG	Anti-inflammatory	HAOEC/THP-1 stimulated with TNF-*α*	1–20 μM, 4–24 h	Inhibit monocyte adhesion and endothelial cell migration	[45]
UA, UB, UC, UD	Anticancer	22Rv1 EROD assay	6.75–50 *μ*M, 0.5, 24 h	Inhibit CYP1B1 and CYP1A1 activity	[46]
UA, UB	Anticancer	Caco-2 cells	40 *μ*M, 24–96 h	Cell cycle arrest in S and G_2_/M phases	[47]
UA	Anticancer	HEK T293 cells Wnt luciferase assay	0.2–200 µg/mL 48 h	Wnt pathway inhibition	[48]
UA, UB, UC, UD	Anticancer	HT-29 cells	25–70 *μ*M	Apoptosis induction and CYP1 activity inhibition	[49]

Caco-2: human colon carcinoma cell line; CCD18-Co: human colon fibroblast cell line; CYP1A1: cytochrome P450, family 1, member A1; CYP1B1: cytochrome P450, family 1, member B1; DCFH-DA: 2′,7′-dichlorodihydrofluorescein diacetate; DMNQ: 2,3-dimethoxy-1,4-naphthoquinone; EROD: ethoxyresorufin-O-deethylase assay; HAOEC: human aortic endothelial cell line; HEK T293: human embryonic kidney cell line; HL-60: promyelocytic leukaemia cells; HT-29 human colon carcinoma cell line; MCF-7: human breast cancer cell line; PGE2: prostaglandin E2; PMA: phorbol 12-myristate-13-acetate; SK-N-MC: human neuroblastoma cells; THP-1: human acute monocytic leukaemia cell line; UA: urolithin A; UAG: urolithin A glucuronide; UB: urolithin B; UBG: urolithin B glucuronide; UC: urolithin C; UD: urolithin D; 22Rv1: human prostate carcinoma cell line.
